# Molecular targets of cannabinoids and their derivatives in epilepsy – a review with focus on CBD

**DOI:** 10.3389/abp.2025.15251

**Published:** 2025-09-30

**Authors:** Sebastian Marciniak, Weronika Wasyluk, Andrzej Wojtak

**Affiliations:** ^1^ Department of Pharmacology, Faculty of Health Sciences, Medical University of Lublin, Lublin, Poland; ^2^ Faculty of Health Sciences, Medical University of Lublin, Lublin, Poland; ^3^ Chair and Department of Vascular Surgery and Angiology, Medical University of Lublin, Lublin, Poland

**Keywords:** cannabinoids, epilepsy, Δ9-THC, CBDV, Δ9-THCA, CBD

## Abstract

In recent years, cannabinoids and their derivatives have been tested for efficacy in epilepsy therapy and related disorders. Many of them may help alleviate ailments associated with seizures. An in-depth study of cannabinoid derivatives and the receptors on which they operate give us a chance for more effective use of these substances in epilepsy therapy. Many studies point to the beneficial synergy of cannabinoids with chemotherapeutics and the increase in effectiveness of the latter. As a result, both alternatives to drug treatment and support for the pharmacotherapy are being developed. In this review, we focused on compounds such as Δ9-THC, CBDV, Δ9-THCA, Δ9-THCV, H2CBD and their receptors as well as on CBD’s actions, and the enzymes, ion channels, and transporters engaged in the fundamental causes of epileptic seizures. Treating epilepsy and drug-resistant epilepsy are the two common medical uses of cannabinoids. We looked at approximately 150 current scientific articles from peer-reviewed journals to explore the molecular effects of cannabinoids in these applications. Our goal was to improve physician awareness of factors influencing treatment decisions and potential adverse reactions to minimize medical errors and optimize patient care.

## Introduction

This review examines cannabinoid derivatives under worldwide research and their molecular targets, focusing on their therapeutic potential in epilepsy. Despite extensive research, many mechanisms underlying the therapeutic effects of cannabinoids remain incompletely understood are not fully appreciated in clinical practice. The primary objective of this review is to provide a comprehensive and critically appraised elucidation of these molecular mechanisms, with a particular emphasis on recent findings, to facilitate their informed and widespread application. Our integrated focus on molecular and clinical evidence provides a distinct contribution by bridging basic science with real-world patient outcomes.

We begin with Δ9-THC, one of the most widely recognized cannabinoids, and then extensively analyze Cannabidiol (CBD), acknowledging the wealth of ongoing and recently completed clinical trials that have led to its regulatory approval for specific epilepsy syndromes. We also explore less-known but promising compounds such as Cannabidivarin (CBDV), Δ9-Tetrahydrocannabinolic acid (Δ9-THCA), Δ9-Tetrahydrocannabivarin (Δ9-THCV), and the synthetic analog 8,9-dihydrocannabidiol (H2CBD). This review integrates the latest scientific developments, encompassing both molecular pharmacology and clinical outcomes, to offer a robust and up-to-date perspective on the role of cannabinoids in epilepsy.

## Materials and methods

A comprehensive narrative review with systematic search elements was conducted to identify and critically appraise the relevant scientific literature on the molecular targets and therapeutic effects of cannabinoids in epilepsy.

### Search strategy

The MEDLINE database via PubMed (United States National Library of Medicine) was systematically searched for articles published up to June 1st, 2025. The primary search strings used were:

1. (“cannabis” OR “cannabinoids” OR “cannabidiol”) AND “epilepsy” (in title/abstract)
2. (“THC” OR “CBD” OR “CBDV” OR “THCA” OR “THCV” OR “H2CBD”) AND (“epilepsy” OR “seizure”) AND (“mechanism” OR “target” OR “receptor” OR “enzyme” OR “channel” OR “transporter”)
3. (“cannabidiol” AND “epilepsy”) AND (“clinical trial” OR “meta-analysis” OR “adverse event”)

### Inclusion and exclusion criteria

Titles and abstracts were initially screened for direct relevance to cannabinoids, epilepsy, and molecular mechanisms or clinical outcomes. Full-text articles were then retrieved for detailed assessment. Original research articles (*in vitro*, *in vivo* animal studies, and human clinical trials, including randomized controlled trials and observational studies) published in English were included. Review articles, commentaries, and editorials were excluded as primary data sources but were used to identify relevant primary research or existing meta-analyses. Studies not directly investigating molecular targets, therapeutic efficacy, or safety in the context of epilepsy were excluded.

### Screening procedures and data extraction

Initial screening of titles and abstracts was performed by three independent reviewers, with any discrepancies resolved through discussion to reach consensus. Full-text articles of all potentially relevant studies were subsequently obtained and meticulously reviewed for their eligibility. Key data extracted included: specific cannabinoid(s) studied; identified molecular targets (receptors, enzymes, ion channels, transporters); proposed mechanisms of action; observed therapeutic or adverse effects; study design (*in vitro*, specific animal model, human clinical trial phase/type); and species (human, mouse, rat, pig). Information on clinical outcomes, such as seizure frequency reduction, responder rates, and specific adverse events, was extracted from clinical trials.

### Study quality appraisal and evidence hierarchy

Given the diverse nature of the included studies (ranging from mechanistic *in vitro* experiments to multi-center clinical trials), a formal quantitative meta-analysis of molecular targets was not performed due to inherent heterogeneity. Instead, a rigorous qualitative critical appraisal was conducted. Evidence was hierarchically considered, prioritizing findings from well-designed human clinical trials (especially randomized, placebo-controlled trials and comprehensive meta-analyses) for clinical efficacy and safety. Mechanistic insights from *in vivo* animal models were considered highly relevant, while *in vitro* studies provided foundational understanding of molecular interactions.

### Grey literature policy

We did not include grey literature in this review, as it has not undergone a peer-review process, ensuring that all cited sources meet scientific publication standards.

## Objectives

To compile and critically evaluate the actions of known cannabis derivatives, specifically identifying which receptors/processes are responsible for these actions, comprehensively assessing the strength of the underlying molecular evidence, and integrating the most recent findings on efficacy and safety in epilepsy.

## Δ9-THC

Δ9-Tetrahydrocannabidiol (Δ9-THC) is one of the best-known cannabinoids (Table 1). This substance is responsible for the psychotropic effects of marijuana. The best-known Δ9-THC targets are the cannabinoid receptors type 1 (CB1) and type 2 (CB2), for which it is a partial agonist (Pertwee et al., 2014). Numerous studies have shown that THC has an anticonvulsant effect or that it modulates the action of antiepileptic drugs (AEDs). However, it should be noted that studies have also been described in which THC had no effect on convulsions, was provocative or the effect was inconclusive (Gaston and Friedman, 2017). The expression of CB receptors was found both in epilepsy in humans and in animal models of epilepsy. Their activation, regardless of the type of transmitter, reduces the release of neurotransmitters, while epileptic activity may be the result of an imbalance between excitatory (E) and inhibitory (I) synaptic transmission (Alger, 2014). In studies in mice it was also shown that the lack of CB1 and CB2 receptors causes epilepsy, which also proves the role of the endocannabinoid system in the regulation of brain excitability (Rowley et al., 2017). The results of other studies suggest a synergistic role of CB signalling in the modulation of early epileptogenic changes and that correlates with CB1R, 5-HT2CR, and NMDAR functions (Di Maio et al., 2019). However, the results of studies on the action of anticonvulsant Δ9-THC are not conclusive. This may result from both the universal inhibitory effects of cannabinoid receptors (they inhibit the release of excitatory and inhibitory transmitters, which makes their total impact on neuronal circuits not easy to predict), as well as from Δ9-THC-pleiotropic effect (affecting various receptors and signalling systems) (Alger, 2014; Gaston and Friedman, 2017). There are also studies suggesting that activation of the endocannabinoid system may be neuroprotective and prevent neuronal damage caused by epileptic seizures.

**TABLE 1:** Chemical structure of the described cannabinoids.

Endocannabinoids	
N-arachidonoylethanolamine (anandamide, AEA)	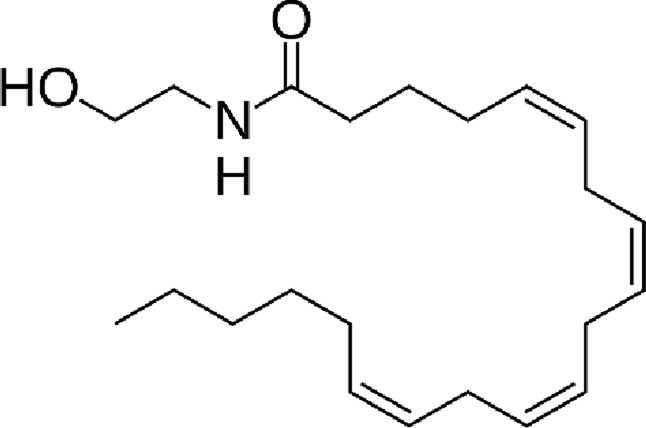
2-Arachidonoylglycerol (2-AG)	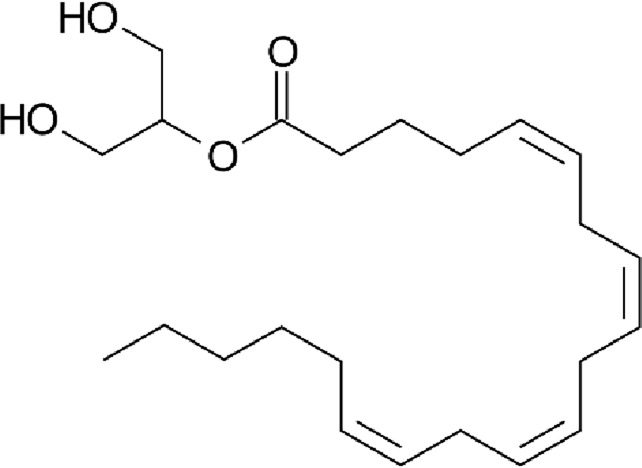

Phytocannabinoids	
Tetrahydrocannabinol (THC)	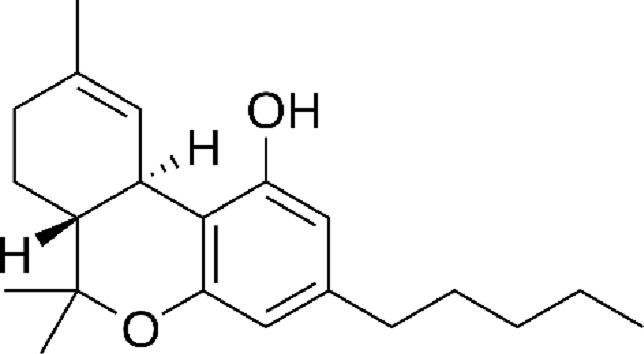
Cannabidiol (CBD)	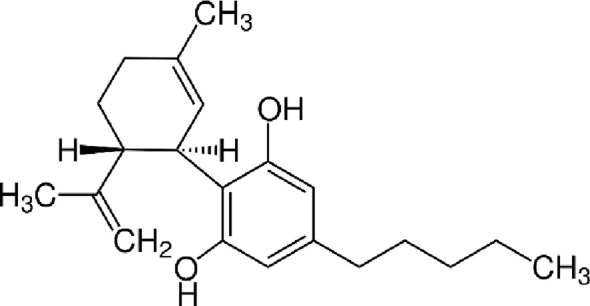
Cannabidivarin (CBDV)	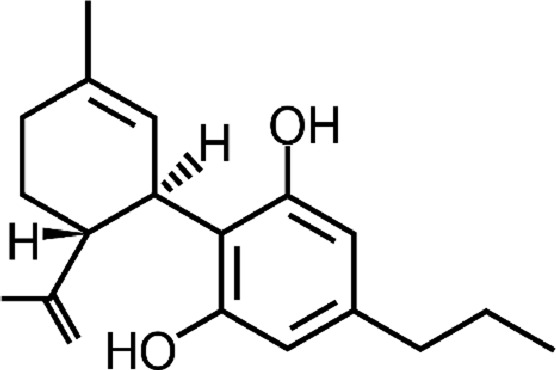
Tetrahydrocannabinolic acid (THCA)	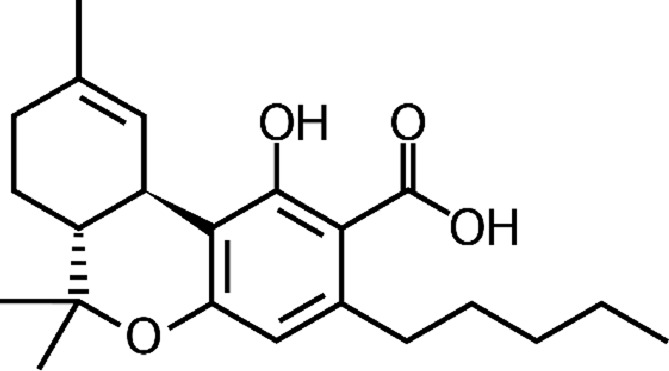
Tetrahydrocannabivarin (THCV)	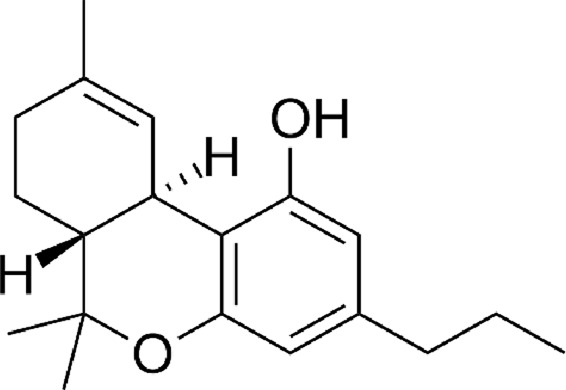

Synthetic cannabinoids	
8,9-dihydrocannabidiol (H2CBD)	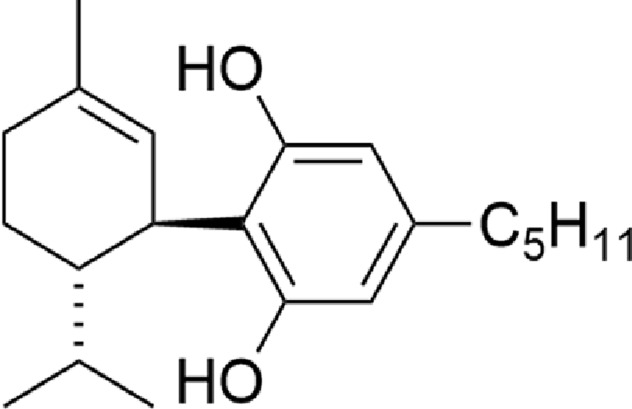

In addition to the relatively well-described effect of THC on cannabinoid receptors, the following receptors are also mentioned in the literature: transient receptor potential (TRP) cation channels (TRPA1, TRPV2, TRPM8), 5-hydroxytryptamine receptor (5-HT3A), opioid receptors (μ, δ), orphan G-coupled protein GPR55 receptor, peroxisome proliferator-activated gamma receptor (PPARγ), β-adrenoreceptors and some ion channels, but the effects of their activation by THC are not fully understood. (Pertwee et al., 2014; Gaston and Friedman, 2017).

## CBD

Cannabidiol (CBD), like Δ9-THC, is a phytocannabinoid compound, but unlike it, has very low affinity for cannabinoid receptors. This lack of affinity for CB1 receptors results in a lack of psychoactivity. It also means that the antiepileptic effects of CBD cannot, as in the case of Δ9-THC, be explained by inhibition of transmitter secretion (McPartland et al., 2015). In studies on an animal model of epilepsy (maximum electrical shock), the efficacy of Δ9-THC, CBD and WIN 55,212-2 in the treatment of seizures has been demonstrated. Using the specific CB1 receptor antagonist (SR141716A), it was proven that the anticonvulsant effect of THC and WIN 55,212-2 is dependent on the CB1 receptor, whereas CBD is independent of it (Wallace et al., 2001). Although the exact CBD anticonvulsant mechanism remains unknown, many molecular targets have been identified in recent years and several potential anticonvulsant mechanisms have been proposed for this compound (Ib et al., 2015). A review of CBD molecular targets described in the literature can be found in Table 2. Due to their role in the cell, they can be divided into receptors, enzymes, ion channels and transporters. It is suggested that the vanilloid receptor from the group of transient potential channels (TRPV1) may participate in anticonvulsant CBD’s activity. TRPV1 is involved in the modulation of epileptic seizures. It is a non-selective channel characterized by significant permeability to calcium ions. Its activation leads to increased release of glutamate and concentration of calcium ions, which results in excitability of neurons (Nazıroglu, 2015). CBD is an agonist of TRPV1 channels, its action causes their desensitization and, as a consequence, normalization of intracellular calcium concentration (Vilela et al., 2017). Another possible mechanism of anticonvulsant CBD’s activity is associated with calcium type T ion channels (T-Type Ca2^+^). Calcium channels are involved in the regulation of neuronal excitability. Activation of these channels is associated with hyperpolarization of the neuronal cell membrane and leads to an increase in the concentration of calcium ions in the cell, which causes excitability. This mechanism is observed in epilepsy (Nelson et al., 2006). CBD blocks T-type calcium channels, which may be responsible for the antiepileptic effect, but there are no studies to confirm (Silvestro et al., 2019). Serotonin (5-HT) receptors may also be important, as they can polarize and depolarize neurons, thereby affecting their conductivity. However, the results of research on their role in epilepsy remain controversial (Gharedaghi et al., 2014). CBD is an agonist of 5-HT1A and 5-HT2A receptors (Russo et al., 2005). The role of these receptors in epilepsy remains unclear, although it is assumed that they may be a therapeutic target for CBD. Opioid (Theodore et al., 2012; Theodore et al., 2007) receptors (ORs) belong to the group of G-protein coupled receptors (GPCR) and are involved in the pathology of some neurological disorders, e.g., epilepsy (Snead, 1986). CBD is an allosteric modulator of μ and δ opioid receptors, which may contribute to the mechanism of its anticonvulsant activity, but this has not been definitively confirmed (Kathmann et al., 2006). CBD is an antagonist of GPR55, which belongs to the receptors involved in the modulation of synaptic transmission (Ryberg et al., 2007). It is an important therapeutic target for CBD, including the Dravet Syndrome (Kaplan et al., 2017). The impact of CBD on cytochrome P450 (CYP450) should also be discussed, although this does not affect the anticonvulsant effect. CBD inhibits hepatic metabolism (Jiang et al., 2013; Yamaori et al., 2011a; Yamaori et al., 2011b; Yamaori et al., 2012; Yamaori et al., 2010; Yamaori et al., 2015; Yamaori et al., 2013), which is important because this cytochrome is involved in the metabolism of some drugs used in epilepsy and can modify their action (Silvestro et al., 2019).

**TABLE 2:** Molecular targets of cannabidiol (CBD) (H–human, M–mouse, R–rat, P–pig).

Molecular target	CBD function	Type of research	Reference
Receptors			
CB_1_ receptor	No significant change	H *in vitro* H *in vitro* H *in vitro*	Ramer et al. (2013) Jenny et al. (2009) Ryberg et al. (2007)
	Negative allosteric modulator	H *in vitro*	Laprairie et al. (2015)
	Activator	H *in vitro*	Sta et al. (2015)
CB_2_ receptor	No significant change	H *in vitro* H *in vitro* H *in vitro*	Ramer et al. (2013) Jenny et al. (2009) Ryberg et al. (2007)
α1 glycine receptor	Activator	H *in vitro*	Ahrens et al. (2009)
α1β glycine receptor	Activator	H *in vitro*	Ahrens et al. (2009)
α3 glycine receptor	Suppresses inflammatory and neuropathic pain by targeting α3 GlyRs	M *in vivo*	Xiong et al. (2012)
GPR3	Inverse agonist	H *in vitro* H *in vitro*	Laun and Song (2017) Laun et al. (2019)
GPR6	Inverse agonist	H *in vitro* H *in vitro*	Laun and Song (2017) Laun et al. (2019)
GPR12	Inverse agonist	H *in vitro* H *in vitro*	Laun et al. (2019) Brown et al. (2017)
GPR18	Partial agonist/antagonist	M *in vivo*	McHugh (2012)
GPR55	Antagonist	M *in vivo* M *in vitro* H *in vitro*	Li et al. (2013) Ryberg et al. (2007)
5-HT_1A_	Agonist	H *in vitro*	Russo et al. (2005)
	Activator	R *in vivo*	De Gregorio et al. (2019)
	Enhances cortical 5-HT/glutamate neurotransmission	M *in vivo*	Linge et al. (2016)
5-HT_2A_	Agonist	H *in vitro*	Russo et al. (2005)
nAChR α-7	Inhibitor	H *in vitro*	Mahgoub et al. (2013)
Opioid receptor δ	Allosteric modulator	R *in vitro*	Kathmann et al. (2006)
Opioid receptor μ	Allosteric modulator	R *in vitro*	Kathmann et al. (2006)
PPARγ	Upregulation/Translocation of PPAR-γ to the nucleus PPAR-γ-dependent apoptotic cell death	H *in vitro*	Ramer et al. (2013)
	Activator	M *in vivo*	Hegde et al. (2015)
Receptor sigma-1 (σ1R)	Antagonist	M *in vivo*	Rodríguez-Muñoz et al. (2018)
NMDA receptor	Inhibitor	M *in vivo*	Rodríguez-Muñoz et al. (2018)
GABAA	Positive allosteric modulator	H *in vitro*	Bakas et al. (2017)
Dopamine D2High receptors	Partial agonist	R *in vitro*	Seeman (2016)
Enzymes			
Acyltransferase acylo-CoA: cholesterol (ACAT)	Inhibitor	H *in vitro*	Cornicelli et al. (1981)
Arylalkylamine N-acetyltransferase (AANAT)	Inhibitor	R *in vitro*	Koch et al. (2006)
Catalase	Inhibitor	M *in vitro*	Usami et al. (2008)
Complex I	Inhibitor	P *in vitro*	Fišar et al. (2014)
	Activator	R *in vivo*	Valvassori et al. (2013)
Complex II	Inhibitor	P *in vitro*	Fišar et al. (2014)
	Activator	R *in vivo*	Valvassori et al. (2013)
Complex II–III	Activator	R *in vivo*	Valvassori et al. (2013)
	Inhibitor	P *in vitro*	Singh et al. (2015)
Complex IV	Activator	R *in vivo*	Valvassori et al. (2013)
	Inhibitor	P *in vitro* P *in vitro*	Fišar et al. (2014) Singh et al. (2015)
COX1	Inhibitor	H *in vitro*	Ruhaak et al. (2011)
COX2	No significant change	H *in vitro*	Massi et al. (2008)
	Inhibitor	H *in vitro*	Ruhaak et al. (2011)
CYP2C19	Inhibitor	H *in vitro*	Jiang et al. (2013)
CYP2D6	Inhibitor	H *in vitro*	Yamaori et al. (2011a)
CYP3A4	Inhibitor	H *in vitro*	Yamaori et al. (2011b)
CYP3A5	Inhibitor	H *in vitro*	Yamaori et al. (2011b)
CYP3A7	Inhibitor	H *in vitro*	Yamaori et al. (2011b)
CYP2C9	Inhibitor	H *in vitro*	Yamaori et al. (2012)
CYP1A1	Inhibitor	H *in vitro* H *in vitro*	Yamaori et al. (2010) Yamaori et al. (2013)
	Induction of expression	H *in vitro*	Yamaori et al. (2015)
CYP1A2	Inhibitor	H *in vitro*	Yamaori et al. (2010)
CYP1B1	Inhibitor	H *in vitro*	Yamaori et al. (2010)
DAGL-α	No significant change	H/R *in vitro*	De Petrocellis et al. (2011)
Fatty-acid amide hydrolase (FAAH)	Inhibitor	H *in vitro* H/R *in vitro*	Bisogno et al. (2001) De Petrocellis et al. (2011)
	Activator	H *in vitro*	Massi et al. (2008)
Glutathione reductase	Inhibitor	M *in vitro*	Usami et al. (2008)
Indoleamine-2,3-dioxygenase (IDO)	Inhibitor	H *in vitro*	Jenny et al. (2009)
LOX-5	Inhibitor	H *in vitro*	Takeda et al. (2009)
	Activator	H *in vitro*	Massi et al. (2008)
LOX-15	Inhibitor	H *in vitro*	Takeda et al. (2009)
	No significant change	H *in vitro*	Massi et al. (2008)
NAD(P)H quinone reductase	Inhibitor	M *in vitro*	Usami et al. (2008)
Phospholipase A2	Activator	H *in vitro*	Evans et al. (1987)
Progesterone 17α-hydroxylase	Inhibitor	R *in vitro*	Watanabe et al. (2005)
Aldose reductase	Inhibitor	H/P *in vitro*	Smeriglio et al. (2018)
Superoxide Dismutase (SOD)	Inhibitor	M *in vitro*	Usami et al. (2008)
Sphingomyelinase	Activator (especially Niemann-Pick’s cells)	H *in vitro*	Burstein et al. (1984)
Testosterone 6α-hydroxylase	Inhibitor	R *in vitro*	Watanabe et al. (2005)
Testosterone 16β-hydroxylase	Inhibitor	R *in vitro*	Watanabe et al. (2005)
Topoisomerase II	No significant change (oxidized CBD – inhibitor)	H *in vitro*	Wilson et al. (2018)
Ion channels			
Cav3.1 T-type	Inhibitor	H *in vitro*	Ross et al. (2008)
Cav3.2 T-type	Inhibitor	H *in vitro*	Ross et al. (2008)
Cav3.3 T-type	Inhibitor	H *in vitro*	Ross et al. (2008)
TRPA1	Activator	R *in vitro* R *in vitro* R *in vitro*	Qin et al. (2008) De Petrocellis et al. (2008) Iannotti et al. (2014)
	Activator Desensitization	H/R *in vitro*	De Petrocellis et al. (2011)
TRPV1	Activator	H *in vitro*	Jenny et al. (2009)
	Activator Desensitization	H/R *in vitro*	De Petrocellis et al. (2011)
	No significant change	R *in vitro*	Qin et al. (2008)
	Activator Desensitization	R *in vitro*	Iannotti et al. (2014)
	Activator	H *in vitro* R *in vivo*	Ligresti et al. (2006) De Gregorio et al. (2019)
TRPV2	Activator	R *in vitro* H *in vitro* H/R *in vitro* R *in vitro*	Qin et al. (2008) Nabissi et al. (2013) De Petrocellis et al. (2011) Iannotti et al. (2014)
TRPV3	Activator	M *in vivo*	De Petrocellis et al. (2012)
TRPV4	Activator	M *in vivo*	De Petrocellis et al. (2012)
TRPM8	Inhibitor/No significant change	R *in vitro*	De Petrocellis et al. (2008)
	Inhibitor	H/R *in vitro*	De Petrocellis et al. (2011)
VDAC1	Inhibitor	M *in vitro*	Rimmerman et al. (2013)
Sodium channels (Nav)	Inhibitor	H *in vitro*	Ghovanloo et al. (2018)
Voltage-gated potassium channel subunit Kv2.1	Inhibitor	H *in vitro*	Ghovanloo et al. (2018)
Ca2^+^-activated K^+^ channels of large conductance (BKCa)	Activator	H *in vitro* M *in situ*	Bondarenko et al. (2018)
Transporters			
ABCC1	Inhibitor	H *in vitro*	Holland et al. (2008)
ABCG2	Inhibitor	M *in vitro*	Holland et al. (2007)
Adenosine uptake (ENT-1)	Inhibitor	R/M *in vivo* M *in vitro*	Pandolfo et al. (2011) Carrier et al. (2006)
Anandamide uptake (AMT)	Inhibitor	H *in vitro* H/R *in vitro*	Jenny et al. (2009) De Petrocellis et al. (2011)
Dopamine uptake	Inhibitor	R/M *in vivo*	Pandolfo et al. (2011)
Glutamate uptake	Inhibitor	R/M *in vivo*	Pandolfo et al. (2011)
Mg2+-ATPase	Inhibitor	R *in vitro*	Gilbert et al. (1977)
Noradrenaline uptake	Inhibitor	R *in vitro*	Coyle and Snyder (1969)
Thymidine uptake	Inhibitor	M *in vitro*	Carrier et al. (2006)

## CBDV

Cannabidivarin (CBDV), also found in cannabis, is a CBD homolog with the sidechain shortened by 2 methylene bridges. Its anticonvulsant activity has been confirmed in preclinical studies *in vitro* and *in vivo* (animal model of epilepsy) (Hill et al., 2012; Hill TD. et al., 2013; Amada et al., 2013). The mechanism of anticonvulsant CBDV activity has not yet been explained, however it seems to be independent of cannabinoid receptors (CBDV, like CBD, has no psychoactive properties). The chemical similarity of CBDV to CBD suggests that these compounds may work similarly (Gaston and Friedman, 2017). CBDV also has agonistic activity at TRPA1, TRPV1 and TRPV2 receptors and antagonistic activity in TRPM8 (De Petrocellis et al., 2011).

## Δ9-THCA

Delta-9-tetrahydrocannabinolic acid (Δ9-THCA) is a THC precursor that occurs in live cannabis. The decarboxylation of THCA to THC occurs under natural conditions in the storage and fermentation of cannabis and under the influence of temperature and light, while the *in vivo* metabolism of Δ9-THCA to Δ9-THC is limited due to its separate metabolic pathways (Moreno-Sanz, 2016). *In vitro*, Δ9-THCA effect on activation of TRPA1, TRPV2 and TRPV4 channels and blocking of TRPV1 and TRPM8 channels has been demonstrated (De Petrocellis et al., 2013). It also inhibits cyclooxygenase (COX 1, COX 2) and diacylglycerol lipase alpha (DLGα), which is an important enzyme in 2-AG biosynthesis (Cascio and Pertwee, 2014; Ruhaak et al., 2011; De Petrocellis et al., 2013). There is no evidence of the ability of THCA to penetrate the CNS after systemic administration or the effect of THCA on cannabinoid receptors (Moreno-Sanz, 2016). Despite online reports suggesting the antiepileptic activities of this compound, in fact, there is no evidence (Gaston and Friedman, 2017).

## Δ9-THCV

Delta-9-tetrahydrocannabivarin (Δ9-THCV) is another cannabinoid found in cannabis. It has been shown to be a partial agonist of the CB1 and CB2 receptors (similar to Δ9-THC). In addition, it has activity on TRPA1, TRPV1-4 and GPR55 receptors (Cascio and Pertwee, 2014). A single study showed anticonvulsant efficacy of THCV in an animal model (Hill TD. et al., 2013).

## H2CBD

The psychoactive properties of THC make the use of cannabinoids, in the treatment of diseases, some legal and social difficulties. In recent years, researchers have focused on CBD, which has no psychoactive effect. However, like other phytocannabinoids, it is a controlled substance in many countries, due to the ease of chemical conversion to THC. Due to these problems, a fully synthetic CBD analogue, 8.9-dihydrocannabidiol (H2CBD), was developed. This compound is produced from non-cannabis precursors and cannot be converted to THC (Mascal et al., 2019). In an animal model of epilepsy (pentylenetetrazole-induced seizures in rats), the effectiveness of H2CBD in reducing the number and severity of seizures has been shown to be comparable to CBD. The mechanism of H2CBD anticonvulsant action is unknown (Mascal et al., 2019).

## Conclusion

Our review of the literature, integrating significant findings up to June 2025, has provided a comprehensive overview of the molecular targets underlying the therapeutic effects of cannabinoids, particularly CBD, in epilepsy. While a multitude of molecular targets have been elucidated through *in vitro* and animal models, the evidence clearly demonstrates the multi-faceted nature of cannabinoid action. Rigorous human clinical trials, especially randomized controlled trials and subsequent meta-analyses, have firmly established the clinical efficacy of CBD for specific drug-resistant epilepsies such as Dravet Syndrome, Lennox-Gastaut Syndrome, and Tuberous Sclerosis Complex.

These clinical advancements underscore that the anticonvulsant activity of phytocannabinoids like CBD, Δ9-THC, Δ8-THC, and Δ9-THCB, is not attributable to a single receptor interaction but rather to a complex modulation of numerous physiological pathways. The concept of the “entourage effect,” suggesting a synergistic interplay of active and inactive botanical molecules in whole plant extracts, warrants further rigorous scientific validation in controlled human trials, as current evidence remains largely observational or preclinical.

Recent years have seen the identification of further molecular targets, including various serotonin receptor subtypes, glycine receptors, α2 adrenergic receptors, voltage-gated calcium channels (VGCCs), and acetylcholine receptors, adding to the complexity of cannabinoid pharmacology. While these interactions suggest broader therapeutic potential, the precise contribution of each target to the overall beneficial effect in epilepsy, and particularly whether cannabinoids exert beneficial effects solely through these newly identified targets, remains a key focus of ongoing research. Furthermore, novel compounds like Δ9-THCB and Δ9-THCP, recently isolated and showing high affinity for CB1 receptors and potent cannabimimetic activity, represent promising tools for future investigations into the pathophysiology and treatment of epilepsy. The continuous elucidation of these molecular targets, coupled with robust clinical translation, will pave the way for more targeted and effective cannabinoid-based therapies.

## Discussion

The therapeutic promise of cannabinoids, particularly cannabidiol (CBD), in the treatment of epilepsy has been substantially confirmed in recent years, leading to its regulatory approval for specific severe forms of epilepsy. While the existing literature provides compelling evidence for the anticonvulsant properties of CBD, several critical aspects warrant in-depth discussion and ongoing scientific scrutiny.

### Efficacy and safety: Clinical outcomes and adverse events

The efficacy of CBD in significantly reducing seizure frequency has been unequivocally demonstrated in multiple large-scale, randomized, placebo-controlled clinical trials, particularly for treatment-resistant epilepsies such as Dravet Syndrome, Lennox-Gastaut Syndrome, and Tuberous Sclerosis Complex (Wu et al., 2022; Gastrop, 2022). Typical seizure reduction rates for CBD range from 30% to over 50% in responder populations, with some patients achieving complete seizure freedom. However, the variability in response among patients remains a critical challenge, highlighting the need for personalized treatment approaches. Factors such as genetic variations (e.g., SCN1A mutations in Dravet syndrome), specific epilepsy syndromes, concomitant antiepileptic medications, and individual pharmacokinetic profiles can profoundly influence CBD’s effectiveness.

While CBD is generally well-tolerated, adverse events (AEs) are common and require careful monitoring, especially in pediatric and polypharmacy patients. The most frequently reported AEs in clinical trials include somnolence, decreased appetite, diarrhea, fatigue, and elevated liver transaminases (ALT and AST) (Pauli et al., 2020). Liver enzyme elevations, often transient and dose-dependent, are particularly noteworthy when CBD is co-administered with valproate or clobazam, due to known CYP450 interactions. This necessitates regular liver function monitoring, as acknowledged in the literature. Long-term safety data are still accumulating, emphasizing the need for ongoing post-marketing surveillance and dedicated research into the sustained effects of CBD on organ systems and brain development in vulnerable populations.

### Mechanisms of action: Disentangling complexity and divergence

The precise mechanisms by which CBD exerts its anticonvulsant effects are complex and polypharmacological, involving interactions with multiple molecular targets as detailed in Table 2. CBD’s engagement with TRPV1, T-type calcium channels (Cav3.1, 3.2, 3.3), 5-HT1A receptors, GPR55, and adenosine uptake (via ENT-1) collectively contribute to its broad therapeutic profile. However, it is crucial to distinguish between hypothetical mechanisms identified in in vitro or animal models and those definitively established in human epilepsy. For instance, while CBD’s blockade of T-type calcium channels is robustly shown in cell-based assays (Ross et al., 2008), its clinical relevance as the primary anticonvulsant pathway in humans remains to be fully elucidated. Conversely, the antagonism of GPR55 and modulation of intracellular calcium via TRPV1 desensitization represent more strongly supported mechanisms directly relevant to neuronal hyperexcitability (Vilela et al., 2017; Kaplan et al., 2017).

The existence of divergent research findings across different groups, particularly in preclinical studies (e.g., conflicting reports on THC’s pro-vs. anticonvulsant effects (Gaston and Friedman, 2017)), underscores the importance of experimental rigor. These discrepancies can often be attributed to variations in cannabinoid purity, formulation, dosing regimens, specific animal models of epilepsy, species differences, and the experimental conditions of *in vitro* assays. A critical appraisal of these factors is essential when interpreting and comparing results, as emphasized in our methodology. The polypharmacology of CBD, while beneficial in terms of broad therapeutic potential, also complicates the prediction of drug interactions and a complete understanding of its side effect profile. Further research using advanced techniques, such as optogenetics, chemogenetics, and *in vivo* electrophysiology, is needed to delineate the primary pathways and identify robust biomarkers predictive of patient response.

### Regulatory and ethical considerations

The varying legal status of CBD globally, despite its FDA/EMA approval for specific epilepsies, continues to impact its accessibility and the conduct of large-scale clinical trials. Regulatory hurdles and the lingering stigma associated with cannabis-derived products can impede both scientific research and clinical integration. Ethical considerations are particularly salient in pediatric epilepsy, where the long-term effects of chronic CBD administration on brain development, cognitive function, and endocrine systems are still under investigation. Balancing the demonstrated clinical benefits against these potential long-term risks, especially in vulnerable pediatric populations, requires ongoing vigilance and robust pharmacovigilance programs. The distinction between pharmaceutical-grade CBD and unregulated CBD products is also a critical regulatory and safety concern, as the latter may contain inconsistent CBD concentrations, impurities, or undeclared cannabinoids.

In conclusion, while CBD now stands as a recognized and effective treatment for specific forms of epilepsy, a deeper, integrated understanding of its comprehensive mechanisms, validated efficacy across diverse populations, and long-term safety profile remains essential. Continued collaborative efforts among scientists, clinicians, and policymakers, coupled with stringent critical appraisal of evidence, will be key to unlocking the full and safe potential of cannabinoids in epilepsy therapy.
